# Devenir périnatal issu des grossesses rapprochées: étude rétrospective marocaine

**DOI:** 10.11604/pamj.2023.44.152.31684

**Published:** 2023-03-30

**Authors:** Latifa Mochhoury, Omaima Changuiti, Mohamed Benfatah, Milouda Chebabe

**Affiliations:** 1 Hassan First University of Settat, Higher Institute of Health Sciences, Laboratory of Health Sciences and Technologies, Settat, Morocco

**Keywords:** Grossesses rapprochées, complications materno-foetales, prévention, Close pregnancies, maternal-fetal complications, prevention

## Abstract

**Introduction:**

l’objectif de de cette étude est de déterminer les complications périnatales associées aux grossesses rapprochées.

**Méthodes:**

il s’agit d’une étude cas-témoin rétrospective menée du 1^er^ juin 2020 au 1^er^ juin 2021 au centre hospitalier provincial de Settat. Au total, 670 patients ont été recrutés. Six cent trente personnes ont été réparties en deux groupes. Un groupe de patientes avec des intervalles intergénésique <9 mois (N = 443) et un groupe témoin >9 mois (N = 187).

**Résultats:**

la prématurité, la dénutrition et l'anémie p<0,05 étaient les principales complications et les principaux facteurs de risque de grossesse imminente étaient l'âge >35 ans (OR = 19,079 (4,98; 73,06) p<0,005) et le milieu rural (OR = 0,468)) (0,28; 0,78) p<0,005), niveau socio-économique bas (OR = 3,465 (2,06; 5,81) p<0,005); absence de prescriptions contraceptives postnatales (OR = 15,77 [7,31; 33,99]; p<0,005); absence d´allaitement avant la grossesse (OR = 49,462 [15,78; 155,03]; p<0,05).

**Conclusion:**

des soins préventifs et ciblés sont nécessaires en matière de planification familiale pour éviter les complications périnatales.

## Abstract (en)

**Introduction:**

the purpose of this study is to provide Moroccan data, study maternal risk factors and identify perinatal complications related to closely-spaced pregnancies.

**Methods:**

we conducted a retrospective case-control study at the Provincial Hospital Center in the city of Settat from June 1, 2020 to June 1, 2021. A total of 670 patients were admitted, but only 630 were included in the study. They were divided into 2 groups: a group of patients whose interpregnancy interval was <9 months (N = 443) and a control group whose interpregnancy interval was >9 months (N = 187).

**Results:**

prematurity, hypotrophy and anemia p<0.05 were the main complications, the main risk factors for closely-spaced pregnancies were age >35 years (OR =19.079 (4.98; 73.06) p < 0.005), coming from a rural area (OR = 0.468 [0.28; 0.78] p < 0.005), having a low socioeconomic status (OR =3.465 [2.06; 5.81]; p < 0.005); the absence of contraceptive prescription in the postpartum period (OR =15.77 [7.31; 33.99]; p < 0.005); and breastfeeding breaks before getting pregnant (OR = 49.462 [15.78; 155.03]; p<0.05).

**Conclusion:**

prevention and specific family planning methods are necessary to avoid perinatal complications.

## Introduction

Plusieurs études ont montré que les femmes qui accouchent à des intervalles très rapprochés présentent un risque plus élevé de complications telles que la prématurité, la mortalité néonatale et le retard de croissance intra-utérin [1,2]. Cependant, ces études ne précisent pas si l'association est due à des antécédents obstétricaux ou des facteurs démographiques. Les femmes dont les intervalles entre les grossesses sont très courts sont prédisposées à un décès périnatal, une augmentation de la prématurité et du retard de croissance intra-utérin a été rapportée [3]. Les saignements irréguliers, l'anémie et les infections post-partum deviennent plus fréquents au cours du troisième trimestre causés par des carences maternelles en fer et en folates [4,5]. Les femmes dont les grossesses sont rapprochées sont souvent défavorisées, plus jeunes, moins qualifiées et moins éduquées [1,2].

Habituellement, la grossesse rapprochée est souvent associée au jeune âge des patientes ou aux facteurs socio-économiques défavorables [1]. Bien qu´ils soient péjoratifs ces éléments ne représentent qu´une partie des facteurs de risque des grossesses rapprochées et ne permettent pas la mise en place d´une prévention efficace au Maroc, aucune étude concernant les grossesses rapprochées et le post-partum n'a été réalisée d´où l´objectif de notre étude: 1) de déterminer si les grossesses rapprochées entraînent des complications materno-fœtales; 2) de confirmer si un délai raisonnable (>9 mois) est convenable après un accouchement pour une nouvelle grossesse; 3) et si la couverture contraceptive en post-partum est très sollicitée.

**Problématique:** quels sont les facteurs favorisants des grossesses rapprochées? Les grossesses rapprochées entrainent-elle plus de complications maternelles, obstétricales et néonatales?

**Hypothèse:** les grossesses rapprochées augmenteraient les complications maternelles, obstétricales et néonatales.

## Méthodes

**Cadre de l'étude:** l'étude a eu lieu à la maternité du centre hospitalier provincial (CHP) Hassan II de la ville de Settat; région du Nord-Est du Maroc, et qui fait partie des régions pauvres du pays. La principale occupation des habitants de la région est l'agriculture et les activités connexes. Le CHP Hassan II est rattaché sur le plan hiérarchique à la délégation du ministère de la santé de Settat, créé en 1964, c´est un hôpital géré selon le mode Services de l'Etat Gérés de Manière Autonome (SEGMA) depuis 1995. Il comprend les disciplines de base suivantes: médecine, chirurgie, gynéco-obstétrique et pédiatrie. Il s'agit de la plus ancienne et de la plus grande maternité de référence dans cette région, qui réalise environ 400 accouchements par mois. Elle comprend un service d'urgence avec 6 salles d'accouchement communes, 2 boxes de consultation, 2 salles d'opération et des chambres d'hospitalisation d'une capacité de 65 lits.

**Type de l´étude:** il s´agit d´une étude rétrospective comparative monocentrique, de type exposé-non exposé au sein du centre hospitalier provincial Settat. L'étude consistait à comparer deux groupes: le groupe des exposés et le groupe des non-exposés: le groupe des exposés était défini par un intervalle entre les deux grossesses ≤ 9 mois entre le début de la première grossesse et le début de la seconde. Cet intervalle a été choisi selon les recommandations de l’Organisation mondiale de la Santé (l'OMS) et du Collège National des Gynécologues et Obstétriciens Français (CNGOF): le groupe des non-exposés correspondait à l'intervalle entre deux grossesses >9 mois entre le début de la première et le début de la seconde. Cet intervalle a été choisi en fonction des données de la littérature. L´étude a été réalisée selon les règles éthiques en vigueur en matière de publication médicale: le secret médical et l´anonymat des patientes ont été respectés.

**Participantes/variables de l'étude:** toutes les patientes ayant une grossesse rapprochée, avec un singleton né vivant, entre le 1^er^ juin 2020 et le 1^er^ juin 2021 ont été recensées via les registres de naissance et les dossiers médicaux de la maternité du centre hospitalier provincial de la ville de Settat selon les critères suivants.

**Critères d´inclusion:** une revue de la littérature sur le sujet a orienté les critères d´inclusion, d´exclusion et les données recueillies pour les groupes «cas» et «témoins». Une grossesse rapprochée était définie dans notre étude par un intervalle inférieur ou égal à 9 mois entre un accouchement et la conception d´une nouvelle grossesse. La date de début de grossesse était définie par la dates des dernières règles et parfois l´échographie. Un groupe de patientes (N=443) a ensuite été apparié avec un groupe témoin dont les patientes ayant accouché deux fois avec un intervalle de temps entre l´accouchement de la première grossesse et la conception de la grossesse suivante supérieure ou égale à 9 mois (N=187).

Nous avons utilisé une grille de recueil de données et nous nous sommes appuyés sur les différents facteurs en lien avec l´intervalle entre les grossesses que la bibliographie nous a permis de mettre en exergue. La grille se divise en 4 parties. La première partie concerne les informations générales sur la patiente, la deuxième détaille le déroulement de la grossesse actuelle, la troisième fait le résumé de l´accouchement et enfin la quatrième traite de l´état néonatal. À partir des dossiers médicaux les données néonatales suivantes ont été collectées: les données maternelles comprenant: l´âge, la situation familiale, la situation socio-professionnelle, le niveau scolaire, la parité, le recours à l´allaitement ainsi que le moyen contraceptif prescrit en post-partum de l´accouchement.

Pour chaque grossesse étudiée; il a été noté: l´existence d´une pathologie obstétricale l´hémorragie en post-partum (perte sanguine supérieure à 500 ml); la présence ou non de pré-éclampsie (définie par la persistance d´une tension artérielle systolique supérieure à 140 mm de Hg et/ou une tension artérielle diastolique supérieure à 90 mm de Hg et une protéinurie supérieure à 0,3g/24h) et l´anémie durant la grossesse (un taux d´hémoglobine inférieur à 10g/dl); le mode d´accouchement par voie basse spontanée ou césarienne; menace d´accouchement prématuré, rupture prématurée des membranes, antécédent de mort fœtal intra-utérin MFIU, aspect du liquide amniotique.

Définitions des différents paramètres utilisés: le niveau d´instruction a été scindé en trois groupes; il est dit bas pour les femmes non scolarisées ou de niveau primaire, moyen pour celles de niveau secondaire et supérieur pour celles qui ont été à l´université. L´origine géographique était classée en deux groupes: urbaine pour les femmes habitant au sein de la ville de Settat et rurale pour celle habitant à plus de 10 km de la ville; activité rémunératrice lorsque la profession maternelle exercée durant la grossesse permet à la femme d´avoir un revenu mensuel. Le revenu mensuel: d´après une étude faite en 2007 par le Haut-commissariat au plan (HPC) du Maroc, on a estimé que si le salaire perçu est inférieur à 3000 dirhams le niveau est bas, si le salaire perçu est supérieur à 3000 dirhams le niveau est moyen.

Le poids de l´enfant; le terme de naissance en semaines d´aménorrhée (SA); score d´Apgar à cinq minutes de vie, le poids du nouveau-né à savoir l´hypotrophie (< 10^e^ percentile) ou macrosomie (> 90^e^ percentile) (courbes de références Association des Utilisateurs de Dossiers Informatisés en Périnatalogie, Obstétrique et Gynécologie (AUDIPOG)) [6] et l´hypothermie néonatale a été retenue en cas de température cutanée inférieure à 35°C. Selon l´Organisation mondiale de la Santé, un enfant est né prématuré si sa naissance a lieu avant 37 semaines d´aménorrhée.

**Critères d´exclusion:** les grossesses arrêtées (fausse couche, mort in utero ou interruption de grossesse), la gémellité et la grande multiparité (définie par plus de trois grossesses avant la grossesse rapprochée). Au final six cent trente (630) dossiers ont été inclus; 20 ont été exclues du fait d´une grossesse non évolutive, 10 pour cause de grande multiparité et 10 pour grossesse gémellaire.

**Analyses statistiques:** les données ont été saisies dans une feuille de calcul Excel, exportées et analysées avec IBM SPSS version 13.0. Les fréquences et les pourcentages ont été calculés pour les variables catégorielles. Moyenne (ou médiane) et écart type des variables continues. Selon une analyse multiple, des variables quantitatives et qualitatives ont été créées à partir des données, qui ont été codifiées pour l’analyse statistique. Des tests du chi carré ont été utilisés pour trouver des associations entre les variables catégorielles. Le test de Kolmogorov-Smirnov a été utilisé pour l’étude de la distribution des variables. Le test de corrélation de Pearson a été effectué pour comprendre les relations entre les variables quantitatives; le test de student pour les échantillons indépendants. Nous avons vérifié la multicolinéarité et la singularité parmi les variables indépendantes; et avons vérifié la normalité, la linéarité et l’homoscédasticité en utilisant des diagrammes de dispersion.

Des analyses de régression logistique bivariées et multivariées ont été réalisées afin d’identifier les facteurs de risque et tester l´association entre variable dépendante ou variable à expliquer qui est l´intervalle entre grossesse (Y) et variables indépendantes ou explicatives (x1, x2, x3,...); y= b0 + b1x1 + b2x2 + b3x3 +…Une valeur de p < 0,05 à un niveau de confiance de 95% a été considérée comme statistiquement significative.

## Résultats

**Figure 1:**
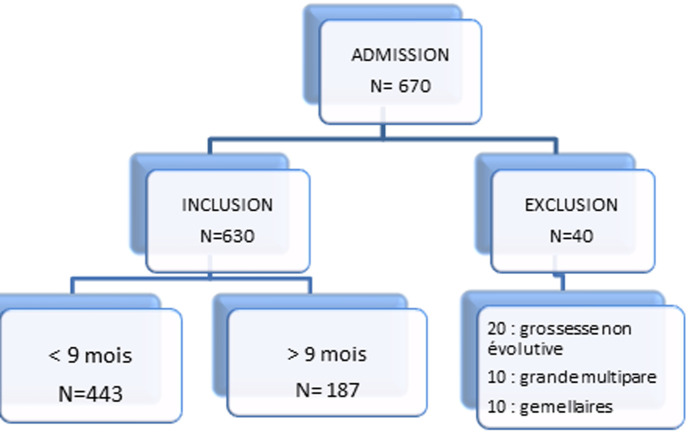
flowchart de la distibution de la population

Six cent trente patientes ont été recrutées et groupées en 2 catégories en fonction des critères d´inclusion et d´exclusion: groupe 1 dont l´intervalle entre grossesse est < 9 mois (N=443) et un groupe témoin dont l´intervalle entre grossesse était > 9 mois (N=187) (Figure 1).

**Caractéristiques de la population étudiée:** l´âge maternel médian était de 32 ans (± 6,59) pour le groupe de patientes dont l´intervalle intergénésique < 9 mois *vs* 29 ans (± 8,40); la différence était significative (p <0,05). Un peu plus de cinquante pour cent (53,7%) des patientes avec une grossesse rapprochée < 9 mois étaient célibataires *vs* 43% chez le groupe témoin (p <0,05) et 34% n´avaient pas de profession contre 6% dans le groupe témoin (p<0,05). En outre 12,6% étaient suivis contre 6% chez le groupe témoin la différence était significative (p<0,05).

La moyenne du terme de naissance était de (36 (±10,3) SA) pour le groupe dont l´intervalle intergénésique < 9 mois *vs* (38 (±3,52)) dont l´intervalle intergénésique >9 mois ainsi le terme < 36 SA était de 53,3% chez la population dont l´intervalle inter génésique < à 9 mois *vs* 26,7% dans le groupe témoin (p<0,001) 89% des patientes avec une grossesse rapprochée < à 9 mois ne bénéficiait pas de moyen contraceptif *vs* 55,6% dans le groupe témoin (p<0,001). Aussi 93% n´ont pas allaité au sein *vs* 57% dans le groupe témoin (p<0,05) (Tableau 1).

**Tableau 1:** caractéristiques maternelles de la population étudiée

Variables		Intervalle entre grossesses		p
		< 9 mois N= 443 ()%	>9 mois N=187 ()%	
Age ans	<21	(46)10,4	(73)39	<0,05
	21-35	(127)28,7	(33)17,6	
	>35	(27) 60,9	(81)43,3	
Etat civil	Mariée	(205)46,3	(112)59,9	<0,05
	Célibataire	(238)53,7	(75)40,1	
Profession	Oui	(292)65,9	(156)83,4	<0,05
	Non	(151)34,1	(31)6,6	
Suivi (consultation)	> 3	(386)87,1	(175)93,6	NS
	<3	(57)12,9	(12)6,4	
Provenance	Rural	(226)51	(72)38,5	0,05
	Urbain	(217)49	(115)61,5	
Niveau socioecono	Bas	(240)54,2	(37)19,8	<0,05
	Moyen	(203)45,8	(150)80,2	
Niveau scolaire	Bas	(212)47,9	(65)34,8 (107)	NS
	Moyen	(197)44,5	57	
	Elevé	(34)7,70	(15)8	
ATCD MFIU	Non	(400)90,3	(174)93	NS
	Oui	(43)9,7	(13)7	
Terme en intervalle en SA	36	(236)53,3	(50) 26,7	<0,05
	36-40	(72)16,3	(114)61	
	40	(13) 30,4	(23)12,3	
Allaitement	Non	(41)293	(108) 57	<0,05
Avant grossesse	Oui	(31)7	(7942)	
Contraception	Non	(396)89,40	(104)55,6	<0,05
Avant grossesse	Oui	(47)10,60	(83)44,40	
Supplémentation en fer	Non	(382)86,2	(166)88,8	NS
	Oui	(61)13,8	(21)11,2	

ATCD MFIU: antécédent fœtal intra-utérine

**Voie d´accouchement:** le recours à la voie haute était statistiquement significatif dans le groupe dont l´intervalle intergénésique < à 9 mois: 79% *vs* 65,8% dans le groupe témoin. La rupture précoce des membranes et l´aspect du liquide teinté étaient majoritaires chez le groupe dont l´intervalle intergénésique < à 9 mois respectivement 55,6% *vs* 9,6% (p < 0,05) et 52,6% *vs* 11,2% (p < 0,05) (Tableau 2).

**Tableau 2:** caractéristiques de l´accouchement chez la population étudiée

Variables		Intervalle entre grossesse		P
		<9 mois N=443 ()%	>9mois N=187 ()%	
Mode	Voie basse	(93)21	(64)34,2	<0,05
Accouchement	Cesarienne	(350)79	(123) 65,8	
Anesthésie	Oui	(304)62,6	(59)41	<0,05
Générale	Non	(182)37,4	(85)59	
Aspect du liquide amniotique	Claire	(210)47	(166)88	<0,05
	Teinte	(233)52,6	(21)11,2	
Rupture des membranes	Pas rupture	(374)84,4	(169)90,4	NS
	Rupture	(69)15,6	(18)9,60	

NS: non significatif, les variables quantitatives ont été exprimées en moyenne ± écart-type et les variables qualitatives en nombres et pourcentages

**Les caractéristiques du nouveau-né:** le poids de naissance moyen était de: (2999g (±1130)) chez les patientes avec une grossesse rapprochée < à 9 mois *vs* (3079g (±786)); 53,3% de nouveau-né prématuré chez les femmes dont l´intervalle intergénésique < à 9 mois *vs* 26,2% (p < 0,001). On notait une différence significative à propos de l´hypotrophie qui était de 50,60% *vs* 24,1%. De même l´incidence de 21,7% de nouveau-né en hypothermie issus d´une grossesse rapprochée < 9 mois *vs* 12,3% chez le groupe témoin (p=0,007).

**Les pathologies associées:** le pourcentage des femmes ayant eu une pré-éclampsie était de 35% *vs* 24% chez le groupe témoin (p < 0,05). L´anémie en cours de grossesse était de 40,9% des patientes avec une grossesse rapprochée < à 9 mois contre 5% dans le groupe témoin (p < 0,05). L´incidence des hémorragies post-partum était de 92,8% chez les patientes avec une grossesse rapprochée < à 9 mois *vs* 57,2% (p < 0,05) (Tableau 3).

**Tableau 3:** caractéristiques du nouveau-né et complications matérno-fœtales

Variables	Intervalle entre grossesse			
		<9 mois N=443 () %	>9 mois N=187 () %	p
Poids catégorie	Hypothrophe	(224) 50,6	(45)24,1	<0,05
	Eutrophe	(72) 16,3	(108)57,8	
	Macrosome	(147) 33,2	(34)18,2	
Sexe	M	(244)55,1	(106)56,7	NS
	F	(199)44,9	(81)43,3	
Score d'apgar 5 min	>7	(339)76,50)	(187)100)	<0,05
	<7	104)23,50)	0	
Mortalité néonatale	Non	(424) 95	(18)97,3	NS
	Oui	(19) 4,3	(5)2,7	
Hypothermie	Non	(347) 78,3	(164)87,7	NS
	Oui	(96) 21,7	(23)12,3	
Prématurité	Non	(206)46,5	(138)73,8	<0,05
	Oui	(237)53,5	(49)26,2	
**Complications maternelles**				
Anémie au cours de la grossesse	Non	(262)59	(176)94	<0,05
	Oui	(181)40,9	(11)5	
Préeclampsie	Non	(287)64	(141)75	0,01
	Oui	(156)35	(46)24	
Hémorragie postpartum	Non	(411)92,8	(107)57,2	<0,05
	Oui	(32)7,2	(80)42, 8	

NS: non significatif, les variables quantitatives ont été exprimées en moyenne ± écart-type et les variables qualitatives en nombres et pourcentages

**Une régression logistique** montre que l´âge> 35 ans était un facteur de risque avec un (OR = 19,07; IC 95 (4.98-75.06); p<0,05); ainsi que le niveau socioéconomique bas et la provenance du milieu rural avec un OR respectif de (OR = 3,46; IC 95 (2.06-5.81); p<0,05) et (OR = 0,46; IC 95 (0.28-0.78); p<0,05) (Tableau 4).

**Tableau 4:** régression logistique multivariée des principaux facteurs de risque des grossesses rapprochées

Délai entre grossesse		B	OR	IC à 95 %	P
<9 mois	Constante	-5,622			0.00
	Age >35 ans	2,949	19,079	[4,98 -73,06]	<0,05
	Milieu rural	-0,76	0,468	[0,28 - 0,78]	0,004
	Niveau socioéconomique bas	1,243	3,465	[2,06 - 5,81]	<0,05
	Pas de contraception précédente	2,758	15,77	[7,31 - 33,99]	<0,05
	Pas d'allaitement précèdent	3,901	49,462	[15,78 -155,03]	<0,05

Le fait de ne pas recourir à un allaitement naturel et ne pas adopter une contraception pendant la grossesse précédente étaient des facteurs de risque avec OR respectif de (OR = 49,46 ; IC 95 (15.78-155.03; p<0,05) et (OR = 15,77; IC 95 (7.31-33.99); p <0,05). On a pu alors établir ainsi une équation logistique une fois sortis les paramètres de la régression (B) et avoir un logarithme de probabilité d´avoir une grossesse rapprochée compte tenu des caractéristiques issues de la régression logistique multivariée (Tableau 4).

Equation de la régression: Y= B0 + B1X1 + B2X2 + B3X3+ B4X4 + B5X5. Y= Log (probabilité de grossesse rapprochée); B0 = -5,622; B1X1= +3,901 (pas d´allaitement); B2X2= +7585 (pas de contraception); B3X3=+1,243 (niveau socioéconomique bas); B4X4= -0,76 (milieu rural); B5X5=+2,949 (âge >35 ans).

## Discussion

Six cent trente femmes ont été incluses; nous avons apporté des données premières sur les grossesses rapprochées au Maroc et avons confirmé qu´un statut socioéconomique bas, l´âge avancé, la non prescription de contraception en postpartum et le non recours à l´allaitement étaient incriminés dans la survenue de complications materno-foetales notamment d´anémie, de prééclampsie, d´hypotrophie et d´hypothermie plus élevée. L´âge moyen était de 32 ans ± 6,59; les patientes du groupe dont l´intervalle entre grossesse < 9 mois étaient plus âgées, près de 60,9% d´entre elles sont âgées de plus de 35 ans contre 43,3% des patientes du groupe témoin; ce qui rejoint la littérature [7,8] et contredit l´étude française de Reims [9] qui a incriminé l´âge de moins de 21 ans comme facteur de risque (multiplié par 20,3). Aussi notre étude rejoint Kaharuza *et al*. qui ont confirmé que l´âge avancé (après 30 ans) était un facteur de risque de grossesses rapprochées au Danemark, à cause de la place occupée par l´activité professionnelle de la femme [10] et la crainte de la ménopause.

En outre les conditions de précarité; le célibat et le niveau scolaire bas pourraient avoir une influence négative sur le suivi de la grossesse, et l´accès aux établissements de soin et donc de rater les programmes instaurés au sein de ces structures à savoir celui de la classe des mères, les consultations prénatales; la planification familiale l´éducation sanitaire et nutritionnelle. Le recours au moyen contraceptif était insatisfaisant et la proportion significativement importante de femmes non allaitantes dont l´intervalle intergénésique <9 mois peut être liée soit à un choix de la patiente qui souhaite une grossesse rapprochée ou à un défaut d´information.

Nos résultats montrent que l´hypotrophie et la prématurité, ont été retrouvées de manière significative comme un risque néonatal 53,5% chez le groupe cas contre 26,2% chez le groupe témoin. Une étude réalisée par Sundtoft *et al*. en 2011, avait rapporté que la concentration du collagène cervical dépend de l´intervalle intergénésique et chute à la suite d'un accouchement [11]; cependant, la normalisation de cet indicateur n'est atteinte que 12 mois après l'accouchement. Par conséquent, il y a intérêt à retarder l'intervalle entre les naissances pour restaurer le tonus musculaire cervical avant le début d'une nouvelle grossesse.

Notre résultat rejoint ceux de l´étude française de Dedecker qui confirme que la prématurité <37 SA était 18,8% chez les mères dont l´intervalle intergénésique chez les patientes < à 9 mois, versus 7,6% dans le groupe témoin (P=0,001) [9]. Une méta-analyse de 2006 de Condé [12] estimait que le risque d'accouchement prématuré était de 1,4 pour les femmes dont les deux grossesses étaient séparées de moins de 6 mois par rapport aux grossesses séparées de 18-23 mois.

En revanche, Zhu [13] a trouvé un risque 1,3 fois plus élevé d'accouchement prématuré chez les mères dont l´intervalle <9 mois (OR = 1,4), et une étude française de Vandenbroucke en 2012 [14] a montré que 17% des nouveau-nés étaient hypotrophes (< 10^e^ centile) à la naissance *vs* 8% dans le groupe témoin (p=0,05) par contre Smits *et al*. [4] ont trouvé dans leur étude un risque 1,3% plus élevé d´hypotrophie pouvant expliquer la survenue de complications, de contraintes physiques et psychologiques, et d'asthénie maternelle lors de la deuxième grossesse.

Notre étude est cohérente avec les études internationales qui ont montré que lors des grossesses rapprochées, les femmes étaient plus anémiées, c´est le cas notamment de l´étude française de Vandenbroucke [14] de 2012, qui avait mis en évidence un risque augmenté de 4,9 d´anémie pendant la grossesse (P=0,001), et un risque augmenté de 2,1 dans le post-partum (P=0,02) dans le groupe grossesses rapprochées (intervalle <9 mois). Ces facteurs sont intimement liés à une carence nutritionnelle en particulier les carences en fer et en acide folique [4, 5, 1,5].

En effet le taux d´acide folique chute dès le cinquième mois de grossesse; le seuil reste très bas pendant plusieurs semaines après l´accouchement par conséquent la survenue d´une autre grossesse est très touchée et prédispose la femme à des risques avant la restauration complète du stock d´acide folique. Certaines prostaglandines, initiatrices de l´accouchement, ont été incriminées. Smits *et al*. supposent en effet une persistance anormalement élevée du taux de ces substances, après le premier accouchement, et les considèrent comme facteurs de risque possibles d´accouchement prématuré [1,6]. Pour cela, lors de suivi de grossesse, les femmes devraient être considérées comme plus à risque d´anémie, une supplémentation en fer et en acide folique doit être souvent nécessaire.

Notre étude n´a pas eu de lien significatif entre les antécédents de mort fœtale intra-utérine MFIU et les grossesses rapprochées contrairement à la littérature [17]. Néanmoins Zhu *et al*. rapportent une association significative entre l´antécédent de mort fœtal intra-utérine (MFIU) et un long délai entre les grossesses, supérieur à 120 mois temps nécessaire aux parents pour faire le deuil de l´enfant perdu [18]. Le groupe avec un intervalle de moins de 9 mois était dominé par l´accouchement par césarienne; nos résultats ont confirmé les résultats de la littérature, ainsi Huang 2002 a montré que les femmes avec des intervalles plus courts entre les grossesses étaient plus susceptibles d'avoir une césarienne [19]; nous avons trouvé une différence significative quant à l'incidence des hémorragies du post-partum; l'étude Condé [12] a confirmé ce résultat, lorsque l'intervalle entre les grossesses était ≤5 mois, le risque augmentait de 70%; la présence de métrorragies s'expliquerait par le nombre augmenté d'anomalies placentaires lors de grossesses rapprochées [20] et un défaut de remodelage des vaisseaux endométriaux [21].

**Limite de l´étude:** le caractère rétrospectif de cette étude constitue sa première limite: les données ont été recueillies uniquement à partir des informations contenues dans les dossiers obstétricaux; la rareté des études nationales concernant ce sujet; dossiers médicaux incomplets; la diversité des résultats entre les différentes études internationales ne permet pas de transposer les résultats d'un pays à l'autre.

**Financement:** cette étude a été financée par le fonds propre de l'Université Hassan First [numéro de subvention FP/2020/02].

## Conclusion

Bien que la définition d'une “grossesse fermée” ne soit pas clairement définie dans la littérature, il est important de ne pas négliger les complications qu'elle peut entraîner. En effet, notre étude a pu montrer que les grossesses rapprochées <9 mois sont associées à plusieurs déterminants (absence de contraception et allaitement artificiel après la première grossesse), âge >35 ans, niveau géographique et socio-économique. Une étude multivariée plus large est nécessaire de toute urgence pour répondre aux recommandations d'espacement entre les grossesses. Notre étude montre que les grossesses rapprochées sont témoins d'une augmentation significative de la vulnérabilité et de la morbidité néonatale (prématurité et hypothermie néonatale). Nous pouvons conclure que les grossesses à un intervalle proche de < 9 mois sont fréquentes et restent préoccupantes en raison de la morbidité périnatale qu'elles génèrent. Une bonne compréhension des déterminants de ce type de grossesse est nécessaire afin d'identifier et d'informer les patientes à risque, ainsi l'importance de la prévention repose sur: la couverture contraceptive post-partum, en particulier lors de l'allaitement; éduquer les femmes en âge de procréer sur les risques d'une grossesse rapprochée et sur le fait qu'un délai raisonnable (> 9 mois) après l'accouchement pour concevoir à nouveau peut réduire le risque; allaitement au sein régulièrement en utilisant la méthode méthode de l'allaitement et de l'aménorrhée (MAMA); supplémentation en fer et en vitamine B9 le plus tôt possible.

### Etat des connaissances sur le sujet

- *Les femmes qui accouchent à des intervalles très rapprochés présentent un risque plus élevé de complications maternelles et néonatales, un décès périnatal élevé, la survenue de la prématurité et le retard de croissance intra-utérin.*

### Contribution de notre étude à la connaissance

- *Données marocaines; déterminer les complications et les facteurs de risques liés à notre société;*
- *Les résultats ont montré des risques maternels et obstétriques importants, notamment en ce qui concerne le risque d'anémie pendant la grossesse et de rupture prématurée des membranes avant 37 semaines;*
- *Les facteurs favorisants de grossesses rapprochées, nous ont permis ainsi de réfléchir à la mise en place d´une prévention adaptée par l´information en suite de couches et la promotion de la contraception; les soins post-partum doivent être envisagés pour les femmes à haut risque d'anémie*.

## Remerciements

Nous souhaitons adresser nos remerciements les plus sincères: au personnel de l´Hôpital Hassan II de Settat et au Pan African Medical Journal d´avoir accepté de réviser ce travail.

## References

[1] Rawlings JS, Rawlings VB, Read JA (1995). Prevalence of low birth weight and preterm delivery in relation to the interval between pregnancies among white and black women. N Engl J Med.

[2] Zhu BP, Rolfs RT, Nangle BE, Horan JM (1999). Effect of the interval between pregnancies on perinatal outcomes. N Engl J Med.

[3] Adams MM, Delaney KM, Stupp PW, McCarthy BJ, Rawlings JS (1997). The relationship of interpregnancy interval to infant bir-thweight and length of gestation among low-risk women, Georgia. Paediatr Perinat Epidemiol.

[4] Smits LJ, Essed GG (2001). Short interpregnancy intervals and unfavourable pregnancy outcome: role of folate depletion. Lancet.

[5] Scholl TO, Johnson WG (2000). Folic acid: influence on the outcome of pregnancy. Am J Clin Nutr.

[6] Mamelle N, Munoz F, Grandjean H (1996). Croissance fœtale à partir de l´étude AUDIPOG. I établissement de courbes de référence. J Gynecol Obstet Biol Reprod (Paris).

[7] Pison G (2010). France 2009 : l´âge moyen de la maternité atteint 30 ans. Popul Soc.

[8] Centers for Disease Control and Prevention (CDC) (1998). Risk factors for short interpregnancy interval--Utah, June 1996-June 1997. MMWR Morb Mortal Wkly Rep.

[9] Dedecker F, Graesslin O, Ceccaldi PF, Baudelot E, Montilla F, Derniaux E (2006). Grossesses rapprochées : facteurs de risque et conséquences périnatales. J Gynecol Obstet Biol Reprod (Paris).

[10] Kaharuza FM, Sabroe S, Basso O (2001). Choice and chance: determinants of short interpregnancy intervals in Denmark. Acta Obstet Gynecol Scand.

[11] Sundtoft I, Sommer S, Uldbjerg N (2011). Cervical collagen concentration within 15 months after delivery. Am J Obstet Gynecol.

[12] Conde-Agudelo A, Belizan JM (2000). Maternal morbidity and mortality associated with interpregnancy interval: cross sectional study. BMJ.

[13] Zhu BP, Le T (2003). Effect of interpregnancy interval on infant low birth weight: a retrospective cohort study using the Michigan maternally linked birth database. Matern Child Health J.

[14] Vandenbroucke L, Lavoué V, Voltzenlogel MC, Le Guellec M, Lassel L, Isly H (2013). Facteurs de risques et conséquences périnatales des grossesses rapprochées: étude cas-témoin rétrospective. J Gynecol Obstet Biol Reprod (Paris).

[15] van Eijsden M, Smits LJ, van der Wal MF, Bonsel GJ (2008). Association between short interpregnancy intervals and term birth weight: the role of folate depletion. Am J Clin Nutr.

[16] Smith GC, Pell JP, Dobbie R (2003). Interpregnancy interval and risk of preterm birth and neonatal death: retrospective cohort study. BMJ.

[17] Polo V, Luna F, Fuster V (2000). Determinants of birth interval in a rural Mediterranean population (La Alpujarra, Spain). Hum Biol.

[18] Zhu BP, Haines KM, Le T, McGrath-Miller K, Boulton ML (2001). Effect of the interval between pregnancies on perinatal outcomes among white and black women. Am J Obstet Gynecol.

[19] Huang WH, Nakashima DK, Rumney PJ, Keegan KA, Chan K (2002). Interdelivery interval and the success of vaginal birth after cesarean delivery. Obstet Gynecol.

[20] Conde-Agudelo A, Rosas-Bermúdez A, Kafury-Goeta AC (2007). Effects of birth spacing on maternal health: a systematic review. Am J Obstet Gynecol.

[21] Razzaque A, Da Vanzo J, Rahman M, Gausia K, Hale L, Khan MA (2005). Pregnancy spacing and maternal morbidity in Matlab, Bangladesh. Int J Gynaecol Obstet.

